# Inferring Heterogeneous Evolutionary Processes Through Time: from Sequence Substitution to Phylogeography

**DOI:** 10.1093/sysbio/syu015

**Published:** 2014-03-12

**Authors:** Filip Bielejec, Philippe Lemey, Guy Baele, Andrew Rambaut, Marc A. Suchard

**Affiliations:** ^1^Department of Microbiology and Immunology, Rega Institute, KU Leuven, Leuven, Belgium;; ^2^Institute of Evolutionary Biology, University of Edinburgh, Edinburgh, United Kingdom;; ^3^Fogarty International Center, National Institutes of Health, Bethesda, MD, USA;; ^4^Departments of Biomathematics and Human Genetics, David Geffen School of Medicine at UCLA, University of California, Los Angeles, CA, 90095, USA;; ^5^Department of Biostatistics, UCLA Fielding School of Public Health, University of California, Los Angeles, CA, 90095, USA

## Abstract

Molecular phylogenetic and phylogeographic reconstructions generally assume time-homogeneous substitution processes. Motivated by computational convenience, this assumption sacrifices biological realism and offers little opportunity to uncover the temporal dynamics in evolutionary histories. Here, we propose an evolutionary approach that explicitly relaxes the time-homogeneity assumption by allowing the specification of different infinitesimal substitution rate matrices across different time intervals, called epochs, along the evolutionary history. We focus on an epoch model implementation in a Bayesian inference framework that offers great modeling flexibility in drawing inference about any discrete data type characterized as a continuous-time Markov chain, including phylogeographic traits. To alleviate the computational burden that the additional temporal heterogeneity imposes, we adopt a massively parallel approach that achieves both fine- and coarse-grain parallelization of the computations across branches that accommodate epoch transitions, making extensive use of graphics processing units. Through synthetic examples, we assess model performance in recovering evolutionary parameters from data generated according to different evolutionary scenarios that comprise different numbers of epochs for both nucleotide and codon substitution processes. We illustrate the usefulness of our inference framework in two different applications to empirical data sets: the selection dynamics on within-host HIV populations throughout infection and the seasonality of global influenza circulation. In both cases, our epoch model captures key features of temporal heterogeneity that remained difficult to test using ad hoc procedures. [Bayesian inference; BEAGLE; BEAST; Epoch Model; phylogeography; Phylogenetics.]

Molecular phylogenetic models typically consider sequence evolution as a continuous-time Markov chain (CTMC) that operates along the branches of a bifurcating tree. As a description of the character substitution process, CTMCs take their values from a finite set of discrete states called the state space and satisfy the Markov property. The Markov property ensures that the process is memoryless, implying that the conditional probability distribution of future states only depends upon the present state, and not on the preceding sequence of events. CTMCs are characterized by matrices of infinitesimal rates that quantify the probabilities of exchanging discrete characters in an infinitely small time interval.

Current CTMC models are not limited to nucleotide or amino acid data, frequently accommodate large state spaces, such as codon substitution models (Goldman and Yang 1994; Muse and Gaut 1994), and generalize to many discrete data types including spatial locations (e.g., cities or countries) in phylogeographic inference (Lemey et al. 2009) or hosts in analyses of viral cross-species transmission (Faria et al. 2013a; Mather et al. 2013). In the latter cases, not only the state space can be large but also the underlying substitution rate matrix may be asymmetric.

Phylogenetic inference often resorts to substitution processes that are stationary, homogeneous, and reversible. Stationarity dictates that the process is at equilibrium, such that the frequency distribution of realized states remains constant over the course of evolution. Homogeneity ensures that the process is constant in pattern throughout evolutionary history, thereby treating evolution as a lineage- or time-independent process. This implies that nonstationarity induces nonhomogeneity, as the process of evolution depends upon the equilibrium frequencies. However, a process can be stationary but not homogeneous, e.g., through the specification of different instantaneous rate parameters for different partitions of the underlying tree topology. Finally, reversibility is a frequently applied restriction to the rate matrix describing molecular evolutionary processes that leads to a reduced number of free parameters. Collectively, these restrictive assumptions make strong abstraction of the underlying substitution process to ease mathematical and computational tractability.

In recent decades, substantial work has aimed at relaxing the standard assumptions in CTMC processes, in order to uncover more complex evolutionary processes and assess their impact on phylogenetic reconstruction. To accommodate nonstationarity, Yang and Roberts (1995), Galtier and Gouy (1998) and Galtier et al. (1999), for example, have proposed models that allow branch-specific nucleotide compositions. Although this includes general treatments involving separate composition parameters for each tree branch, large trees will inevitably lead to over-parameterized models. To address this problem, Foster (2004) has developed an approach that maps a restricted, but fixed number of nucleotide composition vectors with estimable frequencies to the tree. Set in a Bayesian framework, this approach also integrates over the tree topology and finds improved posterior support estimates for topologies in examples with compositional heterogeneity, as opposed to inference under a stationary model that would suffer from attraction artifacts due to similar compositional biases. Further developments have uncoupled compositional shifts from particular nodes in the tree while estimating the total number of events of compositional drift distributed across the tree using a compound Poisson process (Blanquart and Lartillot 2006). Blanquart and Lartillot (2008) have also combined such approaches for amino acid evolution with models that take site-specific substitution patterns induced by protein structure and function into account.

Models also exist to tackle nonhomogeneity in the instantaneous rates of character exchange rather than perturbing stationarity. Homogeneous substitution rates can be relaxed both among sites (Huelsenbeck and Nielsen 1999) and among branches (e.g., Foster et al. 2009), and in both cases this captures additional complexity in the substitution process in different data sets. In codon substitution models, among branch variation in the nonsynonymous to synonymous substitution rate ratio (*d*_*N*_/*d*_*S*_ = ω) allows testing and quantifying varying selective pressure among lineages (Yang 1998).

Tree-based modeling of heterogeneity in the pattern of evolution typically finds its use when applied to widely divergent taxa representing relatively rich speciation histories and possibly involving lineage-specific adaptation. However, a change in the evolutionary process may also apply to an entire population at a particular point in time, in which case the evolutionary shift simultaneously cuts across all lineages at that time point in the underlying genealogy, creating discretized time intervals that we refer to as epochs. Goode et al. (2008) first consider such a scenario; they develop an extension of a codon substitution model with discrete site classes that allows for a time-specific change in ω and in the transition/transversion rate ratio (κ), and estimate probabilities that sites belong to a particular class. Specifying such change-points requires trees measured in time and so Goode et al. (2008) adopt a strict molecular clock model on a fixed tree topology in order to apply the model to HIV envelope sequences sampled from a single patient over a period of three years. Because the rapidly evolving virus population accumulates significant substitutions over such a short time-scale, one can estimate the rate of evolution by incorporating the sampling dates of the sequences (the “dated tip” model, Rambaut 2000). The authors demonstrate that many sites classified as neutral or under positive selection before therapy appear to be under strong negative selection upon treatment initiation.

Although we do not pursue codon substitution models with different site classes in this work, we build upon the approach of Goode et al. (2008) in several important ways. By implementing a similar model of time-specific evolutionary changes in the Bayesian Evolutionary Analysis by Sampling Trees (BEAST) software package (Drummond et al. 2012), we connect the epoch models to different relaxed clock models that often provide a more realistic description of the tempo of evolution (Drummond et al. 2006; Drummond and Suchard 2010). More importantly, we generalize the epoch model to any finite discrete data type and any number of transition times. The former is critical to accommodate discrete phylogeographic inference (Lemey et al. 2009), for which Bahl et al. (2011) recently demonstrate the need to incorporate time-specific migration rates. Our Bayesian approach also does not condition on a fixed tree topology but averages over all plausible evolutionary histories. This integration naturally accounts for uncertainty in the tree and in how the epoch transition times translate to varying branch-specific change points. Jointly estimating the epoch-associated rate matrices and the unknown evolutionary history also ensures that we can fully exploit our Bayesian phylogeographic (or discrete trait evolutionary) inference, which explicitly connects sequence evolution to the trait diffusion process (Lemey et al. 2009). Finally, our implementation also allows us to make use of recent marginal likelihood estimators to assess model fit for different epoch parameterizations (Baele et al. 2012).

Statistical phylogenetic inference can be computationally demanding, especially when confronted with the current flood of sequence data. However, recently new algorithms have been developed to exploit massive parallelization on graphics processing units (GPUs), offering dramatic speed increases for statistical inference under complex evolutionary models (Suchard and Rambaut 2009; Baele and Lemey 2013). By partitioning the time component into discrete intervals, the epoch model further adds to the computational burden, but it also represents an opportunity to exploit massive parallel computation (Suchard and Rambaut 2009; Suchard et al. 2010). To apply the epoch substitution heterogeneity in conjunction with large-state space models to large data sets, we implement our model as part of the Broad-platform Evolutionary Analysis General Likelihood Evaluator (BEAGLE) library for evaluating the likelihood of sequence evolution on trees (Ayres et al. 2012), taking the effort to accommodate multiple scales of parallelization to keep computation time manageable.

Following Goode et al. (2008), we focus mainly on rapidly evolving populations for which significant divergence accumulates between sequences sampled at different time points, both from the simulation perspective and for the real data sets. Using a simulation study we demonstrate that our model can be fit to complex data sets and consistently captures time-specific evolutionary parameters, but is not restricted to time-stamped data. We further demonstrate the use of our model by examining two real-life examples. The first application tests and quantifies changes in *d*_*N*_/*d*_*S*_ associated with HIV disease progression in several different patients. This analysis aims at testing different hypotheses explaining why viral divergence stabilizes close to disease onset in HIV infection (Shankarappa et al. 1999). The second application employs epoch modeling to accommodate seasonality in the inference of global influenza dispersal dynamics.

## Methods

### Time-Homogeneous Substitution Models

Continuous-time Markov chain substitution models provide the cornerstone of computational phylogenetics. Given a discrete trait obtaining *K* distinct states, a *K* × *K* infinitesimal rate matrix **Q** characterizes its CTMC. Matrix **Q** contains instantaneous transition rates *q*_*ij*_ ≥ 0 for *i* ≠ *j* and satisfies **Q1** = **0**, where **1** and **0** are column vectors of size *K*.

From the rate matrix **Q**, a stochastic matrix **P** is computed over time *t* ≥ 0 via matrix exponentiation 
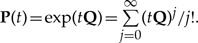
 For an overview of methods to numerically approximate a matrix exponential, we refer to Moler and Loan (1978). Drawing realizations with probabilities defined by **P** gives rise to a stochastic process {*X*(*t*) : *t* ≥ 0} satisfying the Markov property, such that for every *n* ≥ 0, given the time points 0 ≤ *t*_0_ ≤ *t*_1_ < … < *t*_*n*_ ≤ *t*_*n*+1_ and discrete states *i*_0_,*i*_1_,…,*i*_*n*_,*i*_*n*+1_ it holds that *P*{*X*(*t*_*n*+1_) = *i*_*n*+1_∣*X*(*t*_*n*_) = *i*_*n*_,…,*X*(*t*_0_) = *i*_0_} = *P*{*X*(*t*_*n*+1_) = *i*_*n*+1_∣*X*(*t*_*n*_) = *i*_*n*_}. In general, one refers to the elements of **P** as finite-time transition probabilities between the *K* discrete state-space elements. Let us denote a transition probability between two states *i* and *j* over time *u* to *t* + *u* by
(1)
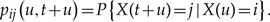


In the phylogenetic setting, researchers often further constrain these processes to be time-homogeneous and time-reversible. Time-homogeneity mandates that transition probabilities depend only on the difference *t* between times *u* and *t* + *u*,
(2)
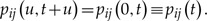

Time-reversible CTMCs satisfy detailed balance, such that π_*i*_p_*ij*_(*t*) = π_*j*_p_*ji*_(*t*) for all *i*, *j* and *t*, where π_*j*_ = *p*_*ij*_(∞) for all *j* return the stationary distribution of the CTMC and are independent of starting state *i*. Finally, common practice in phylogenetics reparametrizes the elements of **Q** into relative rates through the constraint ∑_*i*_π_*i*_*q*_*ii*_ =  − 1 and then, for studies involving phylogenies set in calendar time, multiplies **Q** by a rate scalar *r* to form the argument to **P**(*t*). In this case, we define
(3)
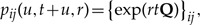

where {·}_*ij*_ extracts the *ij*-th element.

Felsenstein (1981) provides an efficient algorithm for computing the likelihood of a phylogenetic tree **F** given discrete traits and the finite-time transition probabilities along each branch of **F**. Label the nodes *x*_1_,…,*x*_2*N*−1_ in an *N*-tipped **F** set in calendar time. Now, consider a trio of nodes *u*, *v* and *w* where node *u* lies at time *t*_*u*_ in the past and is parent to both nodes *v* and *w*, at times *t*_*v*_ and *t*_*w*_, respectively, in **F**. Then we imagine that an unobserved discrete trait *i* evolves independently into *j* at node *v* over the time interval [*t*_*u*_,*t*_*v*_] with rate scalar *r*_*v*_ and into *k* at node *w* over [*t*_*u*_, *t*_*w*_] with rate scalar *r*_*w*_.

Visiting all the nodes in post-order fashion, we can integrate out these unobserved traits, calculating successive contributions to the partial likelihood for each node via
(4)
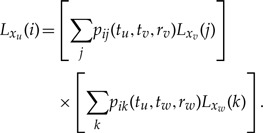


For tip nodes in **F**, we assign 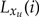 to either 0 or 1 depending on whether trait *i* is (partially) observed or not. Finally, the full likelihood of **F** becomes 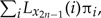 where *x*_2*N*−1_ is the root node. For multiple traits or sequences of length *L* and for among-site rate mixtures with *C* categories, one assumes conditional independence across sites and rate categories and simply aggregates site-category contributions. The serial computational order of this recursion is 𝒪(*K*^2^ × *N* × *C* × *L*).

Central to the recursive tree-pruning in Equation (4) is specification of the branch-specific transition probabilities **P**(*t*_*u*_,*t*_*v*_,*r*_*v*_) = {*p*_*ij*_(*t*_*u*_,*t*_*v*_,*r*_*v*_)} for all *i*,*j*. These are commonly homogeneous and conveniently collapse into functions of just the branch length *t*_*u*_ − *t*_*v*_ instead of the more elaborate starting and ending time. A strict molecular clock assumption specifies that all *r*_*u*_ are equal, but this is not a necessary restriction of our model because we can allow for the introduction of lineage-specific rate variation in addition to the time-heterogeneity in substitution processes that we tackle next.

### Relaxing Time-Homogeneity

The epoch model finds its use in situations where the usual time-homogeneity assumption is violated in specifying **P**(*t*_*u*_,*t*_*v*_,*r*_*v*_). To model nonhomogeneity in process through time, we assume that there exist *S* unique substitution processes characterized through rate matrices **Q**_*s*_ for *s* = 1,…,*S*, and that, at any given point in time, one of these processes is active across all of the extant lineages in **F**. We then model how the active process changes over time via a change-point process with *M* + 1 ordered boundaries at times − ∞ = *T*_0_ < *T*_1_ < ··· < *T*_*M*−1_ < *T*_*M*_ = *t*_*max*_, where *t*_*max*_ is the time of the most recently observed tip in **F**, and *M* indicator functions φ_*m*_ ∊ {1,…,*S*} that identify which 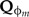 is active during the time epoch [*T*_*m*−1_,*T*_*m*_]. For the examples in this article, we assume that *S*,*M* and (*T*_*m*_,φ_*m*_) for all *m* are fixed through marked biological constraints.

To compute **P**(*t*_*u*_,*t*_*v*_,*r*_*v*_) for each branch in **F** under this change-point process, we return to the Markov property of CTMCs that says one only needs to keep track of the immediate past in determining transition probabilities for the future. This greatly simplifies and regularizes computation, allowing for its parallelization. Assume *t*_*u*_ lies in epoch *m*′ and *t*_*v*_ lies in epoch *m*″. If *m*′ = *m*″, then no new work is necessary. We compute these transition probabilities directly via Equation (3) from an eigen-decomposition of 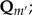 Suchard and Rambaut (2009) describe parallelization of this work across branches and rate categories. On the other hand, if *m*′ ≠ *m*″, the branch traverses *m*″ − *m*′ epoch boundaries at which times **Q** changes. To handle these discontinuities, we imagine a data augmentation procedure to break the nonhomogeneous process into a conditionally independent series of homogeneous processes and then integrate out the augmented data.

**Figure 1.**
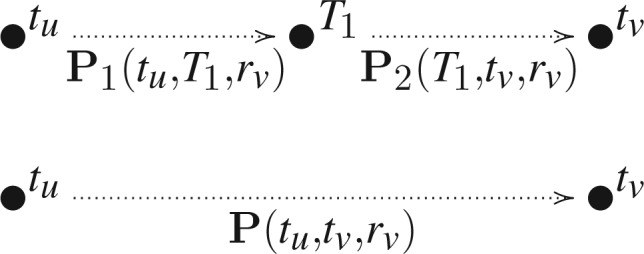
Collapsing branches. Transition probability matrix **P**(*t*_*u*_,*t*_*v*_,*r*_*v*_) governs the nonhomogeneous substitution process along a branch from time *t*_*u*_ to *t*_*v*_ and is the matrix-product of transition matrices **P**_1_(*t*_*u*_,*T*_1_,*r*_*v*_) and **P**_2_(*T*_1_,*t*_*v*_,*r*_*v*_), where *T*_1_ is the epoch change-point time between homogeneous processes 1 and 2. We assume rate scalar *r*_*v*_ remains constant along the entire branch.

Figure 1 illustrates this action for a branch that spans a single boundary at *T*_1_ with **Q**_1_ governing the process before the boundary and **Q**_2_ after the boundary. Letting *X*(*T*_1_) = *k* represent the augmented state of the stochastic process at the boundary, we compute
(5)
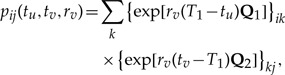

for all *i*,*j*, or equivalently in compact matrix form
(6)
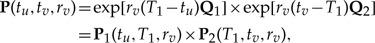

where **P**_1_(*t*_*u*_,*T*_1_,*r*_*v*_) and **P**_2_(*T*_1_,*t*_*v*_,*r*_*v*_) are shorthand notation used in the figure and again in the next section where it is clear that we are considering substitution models for neighboring epochs.

We colloquially refer to the action of Equation (6) as a transition probability matrix convolution to remind the reader that we are integrating out an unobserved state in the middle, but in a strict sense, this action is simply matrix multiplication and exemplifies a Chapman–Kolmogorov equation (see, e.g., Feller, 1968), stating that every stochastic process emitting discrete outcomes as a function of time can be marginalized over one of its variables.

For general *m*′ ≠ *m*″, we arrive at
(7)
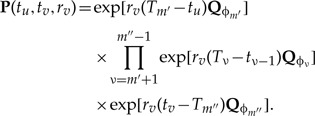


Each matrix convolution in Equation (7) is 𝒪(*K*^3^), potentially commanding a high computational burden compared to the likelihood recursion when *K* is large and many branches in **F** transect multiple boundaries. Fortunately, these operations are very regular and both fine- and coarse-grain parallelization offers a solution to the computational burden.

We implement our epoch model in the BEAGLE library (Ayres et al. 2012) interfaced through the BEAST software package (Drummond et al. 2012). Our BEAST/BEAGLE implementation supports extensive parallel computing on state-of-the-art computer hardware, including GPUs through the Compute Unified Device Architecture (CUDA) framework (Nickolls et al. 2008). In the Supplementary Information (http://dx.doi.org/10.5061/dryad.qp747), we describe our implementation in BEAGLE to achieve efficient fine-scale parallelization, i.e., parallelism where many threads execute small—in the sense of computational complexity and time required for completion—portions of the full task as well as the coarse-grain parallel implementation in BEAST, where a handful of threads are responsible for executing relatively complex and time-consuming tasks.

## Results

### Performance Assessment using Simulation

To evaluate the performance of the epoch model, we conduct a simulation study, in which replicate data are generated along an evolutionary history inferred from a real data set with samples collected at different points in time. Specifically, we use a maximum clade credibility (MCC) tree summarizing a Bayesian phylogenetic inference of human influenza A hemagglutinin gene sequences sampled through different epidemic seasons (Drummond and Suchard 2010).

**Figure 2.**
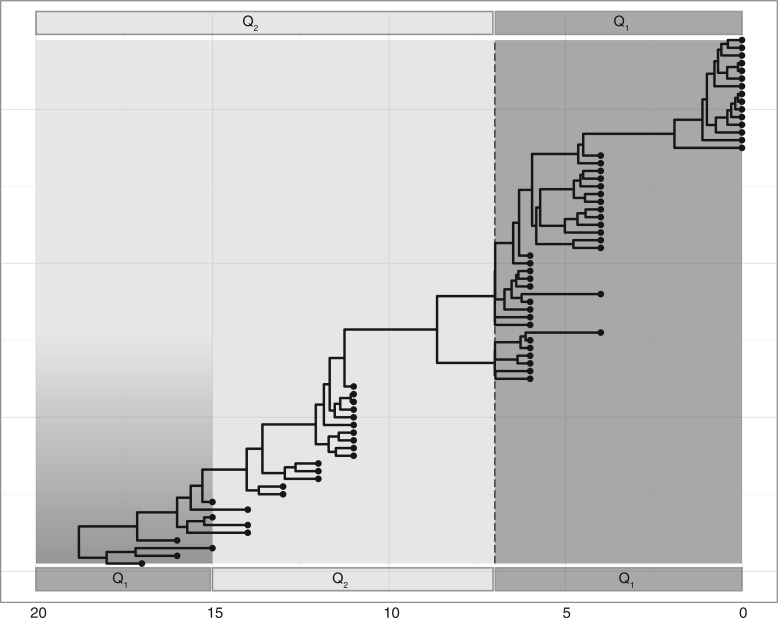
Epoch simulation scenarios on an influenza A maximum clade credibility tree topology. In the two-epoch example illustrated at the top, the transition time is set at *t*_1_ = 7, creating two epochs with substitution processes governed by infinitesimal rate matrices **Q**_**1**_ and **Q**_**2**_ respectively, separated by the light and dark gray areas and the dotted line. In the three-epoch example illustrated at the bottom, transition times are put at *t*_1_ = 7 and *t*_2_ = 15, creating three epochs with substitution processes governed by infinitesimal rate matrices **Q**_**1**_, **Q**_**2**_ and then again **Q**_**1**_, as indicated by the alternating dark and light areas.

In Figure 2, we illustrate the tree topology and the transition times defined for both a two-epoch and three-epoch specification. This tree has 69 tips, is rooted and time-scaled, effectively covering a period of about 18 years. For each replicate data set, we simulate 1000 nucleotide sites under the Hasegawa, Kishino, and Yano (HKY) model (Hasegawa et al. 1985). We set the substitution rates and base frequencies to values estimated from the real data set.

In a first scenario, we test whether the epoch model correctly identifies a homogeneous nucleotide substitution process. We simulate an alignment evolving under the HKY model with a κ parameter (the transition–transversion bias) value of 10.0 for the whole timespan of the tree. In the analyses of replicate data, we specify a boundary time *T*_1_ = 7.0 (7 years before the most recent sampling date) creating *M* = 3 ordered boundaries with *S* = 2 substitution processes governing character changes between them. We then run an MCMC chain, starting from a randomly generated tree topology and assuming proper log-normal priors on parameters κ_1_ and κ_2_. We repeat the simulation and inference process 100 times and report estimator coverage, mean (derived from the estimated values) and mean squared error (MSE) in Table 1.

**Table 1.** Estimator performance for simulated data sets

Simulated		Estimated								
		Coverage	Mean	MSE^a^	Coverage	Mean	MSE	Coverage	Mean	MSE
Nucleotides		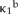			κ_2_			κ_3_		
	κ_1_ = 10	0.96	10.068	1.005	0.98	10.283	1.097	—	—	—
Dated tips	κ_1_ = 1, κ_2_ = 10	0.98	1.007	0.008	0.96	10.446	1.551	—	—	—
	κ_1_ = 1, κ_2_ = 10, κ_3_ = 1	0.96	1.010	0.009	0.96	9.993	2.435	0.95	1.017	0.026
Contemporaneous	κ_1_ = 1, κ_2_ = 10, κ_3_ = 1	0.96	1.049	0.002	0.95	10.002	3.723	0.92	1.022	0.061
codon		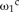			ω_2_			ω_3_		
	ω_1_ = 1	0.94	0.993	0.048	0.93	1.014	0.53	—	—	—
Dated tips	ω_1_ = 0.1, ω_2_ = 1	0.90	0.103	0.001	0.93	1.011	0.054	—	—	—
	ω_1_ = 0.1, ω_2_ = 1, ω_3_ = 0.1	0.92	0.102	0.001	0.89	1.096	0.274	0.96	0.110	0.02
Contemporaneous	ω_1_ = 0.1, ω_2_ = 1, ω_3_ = 0.1	0.93	0.100	0.001	0.96	1.067	0.051	0.95	0.103	0.002

Notes: The table lists the parameter values used to generate data in the first major column and coverage of their estimates, along with measures of variance and bias, in the second major column. Consecutive rows present the results for the first, second, and third nucleotide model simulation for dated-tip samples and the third nucleotide model simulation for contemporaneous sequences (ultrametric tree), followed by the the results of first, second, and third codon model simulation for dated-tip samples and the third codon model simulation for contemporaneous sequences.

^a^Mean Squared Error.

^b^HKY model's transition-transversion bias parameters.

^c^Yang codon model's nonsynonymous to synonymous substitution rate ratio.

Estimator coverage reflects the probability that the true value from which the data derive falls within the model estimated nominal credible interval and hence predicts the performance of the methods across a wide ensemble of data sets. While Bayesian credible intervals do not need to yield nominal coverage, we still obtain coverages of 96% and 98% for κ_1_ and κ_2_, respectively.

In a second simulation scenario, we consider a heterogeneous substitution process in which the recent substitution history (more recent than *T*_1_ = 7.0) is governed by an HKY model with κ = 1.0, and alters to an HKY model with κ = 10.0 beyond that boundary time. By analyzing 100 simulation replicates generated under these settings, we arrive at a coverage of 98% for κ_1_ and 96% for κ_2_.

In a third nucleotide simulation scenario, we consider *S* = 3 epochs, where before time *T*_1_ = 7.0 substitutions occur under an HKY model with a κ value of 1.0, between *T*_1_ and *T*_2_ = 15.0 under an HKY model with κ = 10.0 and after *T*_2_ again under an HKY model with κ = 1.0. The resulting coverages are 95%, 95%, and 89% for κ_1_, κ_2_, and κ_3_ respectively. As we introduce more epochs, we observe a concomitant increase in MSE. This can be expected as partitioning the time into more intervals will typically leave corresponding epochs less informed as less branch length is located in each epoch. For the same value of κ = 1.0 in the three-epoch model, the MSE is somewhat higher for the oldest epoch (0.071) compared to the most recent epoch (0.021), which is also in line with more branch length informing the latter (Fig. 2).

Epoch models are not restricted to nucleotide models; they can also relax time-homogeneity in full codon substitution models, such as the Goldman–Yang (GY94) codon model (Goldman and Yang 1994). We here examine the performance of such codon models in an epoch setting. As before, we first test a homogeneous substitution scenario and check whether the model is able to recover homogeneous values for the ω parameters across epochs. To this end, we simulate 500 nucleotide triplets under the GY94 codon model with an ω parameter value of 1.0. Performing 100 simulation replicates yields a coverage of 94% for ω_1_ and 93% for ω_2_. To asses the coverage in a heterogeneous codon substitution scenario, we set the true values to ω_1_ = 0.1 and ω_2_ = 1.0, with a transition time *T*_1_ = 7.0 between the epochs, which results in a coverage of 90% and 93% for ω_1_ and ω_2_, respectively. We observe a somewhat higher MSE under the homogeneous scenario for ω_2_ despite the fact that we use the same value for both homogeneous and heterogeneous simulations in this case (ω_2_ = 1.0), but the coverage is the same for both simulation scenarios (93%). This highlights the importance of considering the uncertainty of point estimates when assessing potential differences between epoch parameters.

Analogous to the nucleotide simulations, we also asses the epoch model performance when the data are simulated over three heterogeneous epochs, with sequences evolving under the GY94 codon model with ω_1_ = 0.1 before *T*_1_ = 7.0, then with ω_2_ = 1.0 and after time *T*_2_ = 15.0 with ω_3_ = 0.1. We obtain a coverage of 92%, 89% and 96% for ω_1_, ω_2_ and ω_3_ respectively. Also in this case the MSE is higher for the oldest epoch compared to the most recent epoch.

For both nucleotide and codon models we also explore how well epoch parameters can be recovered from contemporaneous sequence data, without sequences sampled throughout the past epochs. To this end, we set all sampling dates to time *t* = 0, effectively transforming the tree topology to be ultrametric (all tips at equal distance from the root, Supplementary Information, http://dx.doi.org/10.5061/dryad.qp747). We list the results for these simulations under the rows labeled as “contemporaneous” in Table 1. The resulting coverages for contemporaneously sampled sequences are 96%, 95% and 92% for κ_1_, κ_2_, and κ_3_ respectively and 93%, 96%, and 95% for ω_1_, ω_2_, and ω_3_, respectively. We note that the MSE is generally lower for estimates produced for the contemporaneous data because the ultrametric transformation implies that more branches inform the epochs.

### Within-Host HIV Selection Dynamics

We re-analyze within-host HIV-1 sequence data from eight patients extensively sampled throughout infection starting close to the time of seroconversion (Shankarappa et al. 1999). These patients have previously been classified as moderate or slow progressors based on progression time, or the time it takes for CD4 + T cell counts to drop below 200 cells/μl (Williamson 2003). The data consist of *env* C2V5 sequences collected over a 6–13.7-year period with an average of 12 time points per patient (see Supplementary Information). The original investigation of HIV-1 diversity and divergence over time in these patients reveals a consistent pattern of divergence stabilization at late-stage infection (Shankarappa et al. 1999). This has led to two different hypotheses that may explain these patterns. The immune relaxation hypothesis posits that the damaged immune system during the symptomatic stage leads to reduced selection pressure on the virus, which relaxes the need for fixing immune escape mutations in the viral population. The cellular exhaustion hypothesis, on the other hand, states that the decreased target cell availability in late-stage infection provides less opportunity for viral replication. While the former only impacts nonsynonymous changes, the latter is expected to reduce both synonymous and nonsynonymous rates of substitutions.

To distinguish between these hypotheses, we ask whether ω decreases at late-stage infection, as defined by the progression time for each patient. This rate ratio is an explicit parameter of the GY94 codon substitution model (Goldman and Yang 1994), which we can extend with an epoch specification. For each patient, we compare a standard homogeneous model to a two-epoch specification with a separate GY94 model before and after boundary time *T*_1_ set to progression time for that patient. We exclude patient 11 from the original study because no sequence data are available after progression time for this patient (Shankarappa et al. 1999). The two-epoch discretization allows estimating a separate ω parameter for the two infection stages in each patient, with ω_2_ denoting the *d*_*N*_/*d*_*S*_ ratio before progression and ω_1_ denoting the same parameter after progression.

**Figure 3.**
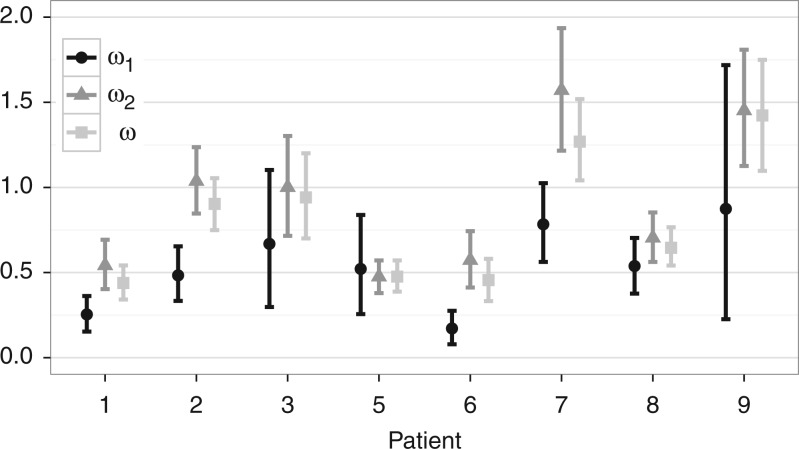
Estimates of *d*_*N*_/*d*_*S*_ ratio for within-host HIV analyses. Vertical lines represent 95% highest posterior density intervals for the *d*_*N*_/*d*_*S*_ ratio estimates. Parameter ω is estimated under the homogeneous model, while ω_1_ and ω_2_ are obtained using the epoch model.

Figure 3 presents the results for the ω parameter estimates. The ω estimates indicate a general decrease in *d*_*N*_/*d*_*S*_ after progression time (ω_1_ < ω_2_, Fig. 3). The most pronounced differences in ω before and after progression time can be observed for patients 1, 2, 6, and 7. For patients 2, 3, 7, and 9, the drop in mean ω estimates suggests a shift in neutral or even positive selection (ω_2_ ≥ 1) to negative selection (ω_1_ < 1). The homogeneous ω estimate is generally closer to ω_2_, which can be expected because most evolutionary history takes place prior to progression time.

Despite the observation that the Bayesian credible intervals for patient 1, 2, 6, and 7 estimates do not overlap, this does not provide a formal test to evaluate their differences. Therefore, we conduct a Bayes factor (BF) test (Suchard et al. 2005) that expresses the posterior odds over the prior odds that ω_1_ < ω_2_ for the individual analyses of each patient. To determine the posterior odds, we note that the MCMC sample average of an indicator function that the parameter values fall within one competing model space converges to the posterior probability of that model. The prior odds in our case is simply 1. The log Bayes factors listed in Table 2 suggest generally strong evidence for a declining selective pressure after progression, with one notable exception for patient 5. We also provide a Bayes factor that summarizes the joint evidence for ω_1_ < ω_2_, which suggest an overall support in favor of the immune relaxation hypothesis (log BF = 2.14), in accordance with previous findings suggesting a general decrease in nonsynonymous divergence at late-stage infection (Williamson et al. 2005; Lemey et al. 2007).

**Table 2.** Bayes factor test for decreased selection after progression

Patient	Posterior probability	log Bayes factor
Patient 1	>0.999	7.418
Patient 2	>0.999	9.602
Patient 3	0.898	2.174
Patient 5	0.430	−0.282
Patient 6	>0.999	9.210
Patient 7	>0.999	8.112
Patient 8	0.933	2.627
Patient 9	0.895	2.142
**Joint evidence:**	0.894	2.14

Notes: We report the posterior probability that ω_1_ < ω_2_ and the corresponding Bayes factor against the alternative that ω_1_ ≥ ω_2_.

### Seasonal Circulation Dynamics of Human Influenza A

In a second application of the epoch model, we focus on discrete diffusion processes to infer spatio-temporal history from viral gene sequences. This type of phylogeographic inference, where the sampling locations are considered as discrete geographic traits, has gained popularity in recent years, at least partly because of a flexible and efficient Bayesian implementation that connects dispersal dynamics to sequence evolution in time-measured phylogenies (Lemey et al. 2009). Recently Bahl et al. (2011) have applied this Bayesian inference framework to investigate the circulation dynamics of global influenza A H3N2 through time. Since the authors were interested in capturing the heterogeneity in these dynamics over successive seasonal epidemics between 2003 and 2006, they consider discrete traits that are the product of sampling location and sampling time (epidemic season). Not only does this discretization by sampling time seem counterintuitive for a model that emits discrete outcomes as a continuous function of time, it also considerably increases the dimensionality of the CTMC rate matrix and thus the number of parameters to inform by the sparse spatial data.

**Figure 4.**
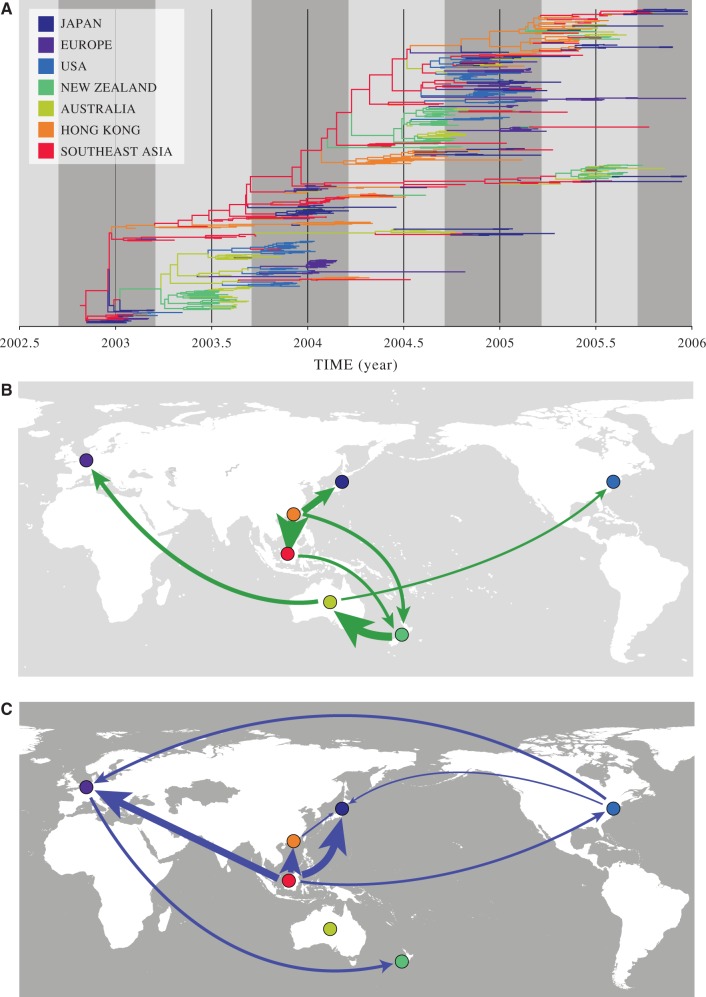
A two-epoch phylogeographic model applied to seasonal influenza H3N2. A. Maximum clade credibility (MCC) tree with branches colored according to modal discrete location states at each node. The gray time intervals represent the epoch model with a single discrete rate matrix shared across northern hemisphere spring and summer (light gray) time intervals and another rate matrix shared across the northern hemisphere autumn and winter (dark gray) time intervals. B. Diffusion rates supported by a Bayes factor >20 for spring and summer epoch intervals. The width of the arrows reflects the magnitude of the Bayes factor support. C. Diffusion rates supported by a Bayes factor >20 for autumn and winter epoch intervals.

Here, we explore epoch time-discretization as a more appropriate alternative to detect temporal heterogeneity in influenza dispersal. We revisit the Bahl et al. (2011) data set that consists of 525 influenza A H3N2 hemagglutinin sequences sampled from Australia, Europe, Japan, New York, New Zealand, Southeast Asia, and Hong Kong (*n* = 75 each) from 2003 to 2006. In a first epoch model extension of the discrete phylogeographic approach, we specify alternating epochs for the time intervals encompassing northern hemisphere spring and summer and the time intervals encompassing northern hemisphere autumn and winter. The discrete diffusion parameters are shared across rate matrices for the spring and summer epochs as well as for the autumn and winter epochs, effectively producing two rate matrices compared to a single matrix for the homogeneous model. Figure 4 schematically represents this *S* = 7 epoch parameterization. Following Kass and Raftery (1995) we report rates that yield a Bayes factor support interpreted as ‘strong evidence’.

We apply a Bayesian stochastic search variable selection (BSSVS) procedure to identify the best supported diffusion rates within each epoch using a Bayes factor test, as available in the SPREAD software (Bielejec et al. 2011). Rates yielding a Bayes factor over 20 are represented in Figure 4A and B for the spring and summer epoch, and autumn and winter epoch, respectively. This suggests seasonal dynamics with spring and summer circulation to a large extent mirroring autumn and winter circulation. The spring and summer epoch appears to be dominated by circulation from Southeast Asia and Hong Kong to the Southern hemisphere (New Zealand), circulation within the Southern hemisphere and also circulation from the Southern to the Northern hemisphere. During the autumn and winter epoch on the other hand, we infer mostly circulation from Southeast Asia to the Northern hemisphere, circulation within the Northern hemisphere and occasional circulation from the Northern to the Southern hemisphere.

To evaluate the improvement of explicitly modeling these largely opposing dynamics, we compared model fit with a homogeneous model using path sampling and stepping-stone sampling, two reliable estimators of marginal likelihood (Baele et al. 2012). Proper priors were used for all parameters during the various analyses, as well as the model selection, since such priors have been shown to be essential when performing marginal likelihood estimation (Baele et al. 2013). The results of the model comparison are listed in Table 3 and provide evidence for the two-epoch model outperforming the homogeneous model. When we further extend our phylogeographic epoch time-discretization to four epochs, modeling separate dynamics for each individual season, we observe additional improvements in terms of marginal likelihoods but with diminishing returns with respect to the two-epoch vs. homogeneous comparison (see Supplementary Information for a visual summary of well supported circulation rates in each season).

**Table 3.** Marginal likelihood estimates

	Marginal likelihood	
Model	PS	SS
homogeneous	− 827.29	− 825.07
7-epoch	− 806.36	− 803.40
14-epoch	− 798.77	− 795.63

Notes: Comparison in terms of model fit between a homogeneous model, an epoch model with time discretized into *S* = 7 epochs alternating between 2 different rate matrices and an epoch model with time discretized into *S* = 14 epochs, alternating between 4 separate rate matrices. PS, path sampling; SS, stepping stone sampling.

## Discussion and Conclusions

Goode et al. (2008) demonstrate that a change in evolutionary pattern affecting all individuals of a population can be modeled by specifying different substitution models across different time intervals rather than over different lineages. Here, we extend this approach and further demonstrate how epoch modeling can uncover temporal heterogeneity in discrete character evolution in phylogenetic histories. We are mainly interested in heterogeneity resulting from variation in the relative intensities of substitutions across time and not heterogeneity induced by nonstationarity. We embed the epoch model in a Bayesian phylogenetic framework that focuses entirely on time-measured trees and integrates over all plausible evolutionary histories for the observed sequence data. Our simulations show that the model is able to recover different scenarios of heterogeneity under different substitution models, but epoch parameters can also reflect an underlying process that is in fact homogeneous, thus avoiding false positives (see Table 1). Following Goode et al. (2008), we primarily focus on time-stamped sequence data from rapidly evolving pathogens for which the ecological and evolutionary dynamics occur on the same time scale and potentially interact. However, our approach does not necessarily require sequence data sampled throughout different epochs. In fact, the simulation study demonstrates that the epoch model can also capture time-heterogeneity in the substitution process inferred from contemporaneous sequence data. In this case, the amount of evolutionary history, as measured by branch length within each epoch, determines how accurately epoch parameters can be inferred.

We apply our model both in the context of sequence evolution and spatial dispersal dynamics. For the former, we focus on within-host HIV-1 evolution and explicitly test different hypotheses that explain the stabilization in sequence divergence in late-stage infection (Shankarappa et al. 1999). This phenomenon has been attributed to weakened selection pressure (immune relaxation) or to a decrease in average viral replication rate (cellular exhaustion) (Williamson et al. 2005). Various studies have attempted to distinguish between both scenarios by contrasting the accumulation of nonsynonymous and synonymous substitutions using different methodologies (Williamson et al. 2005; Lemey et al. 2007; Lee et al. 2008). While two studies provide strong support for the immune relaxation hypothesis using different methodologies (Williamson et al. 2005; Lemey et al. 2007), Lee et al. (2008) suggest that both synonymous and nonsynonymous evolutionary rates decline as disease progresses. Here, we explicitly model a change in the *d*_*N*_/*d*_*S*_ ratio in codon substitution models while integrating over the underlying within-host HIV-1 phylogeny, and formally evaluate the support for a decrease in *d*_*N*_/*d*_*S*_ using Bayes factors. This demonstrates strong overall support in favour of the immune relaxation hypothesis.

Although the hypothesis we test here does not require investigating site-specific selection patterns, we note that codon models that accommodate different categories of sites also have interesting applications in the epoch framework. This has been demonstrated by Goode et al. (2008), who employed a particular codon parameterization to allow for sites to switch among a neutrally evolving class, a class of negatively selected sites and a class of positively selected sites (cf. M2, Nielsen and Yang 1998) before and after the start of HIV-1 antiretroviral therapy. Using this approach, the authors showed that a considerable number of sites, which are identified as neutral under a time-homogenous model, are under some form of selection in one of the two epoch time periods, implying that neutrality may be context-dependent. Codon models that accommodate different categories of sites have not yet been implemented in BEAST, at least partly because they are computationally prohibitive. Incorporating such models in an epoch approach would contribute even more to the computational burden. We hope however that the parallel implementations we pursue here may still allow practitioners to explore such extensions in future research.

Our second application exemplifies the use of epoch time-discretization in phylogeographic inferences that consider sampling locations as discrete traits. In particular, we demonstrate how epoch modeling can capture seasonality in human influenza A H3N2 circulation dynamics, without the need to complicate location traits with sampling time. Based on a data set previously analyzed by Bahl et al. (2011), we infer different epidemiological connections between the northern hemisphere spring-summer epoch and the autumn–winter epoch.

In both cases Southeast Asia (and Hong Kong) appear to play a central role in seeding the seasonal epidemics in the different hemispheres (Fig. 4). However, we remain cautious in interpreting the support for diffusion rates in the context of source-sink dynamics because strong evidence for such a rate being nonzero does not necessarily imply that the diffusion rate itself is high (Faria et al. 2013b). For example, the connection we identify between Europe and New Zealand during the northern hemisphere autumn and winter epidemic may represent a few introductions into New Zealand without extensive onwards transmission. Therefore, it remains difficult to assess which rates govern potential source-sink dynamics.

The specification of two alternating epochs yields a better model fit than a homogeneous model while providing a more parsimonious parameterization compared to the use of discrete traits that are based on both sampling location and epidemic season as in Bahl et al. (2011). Model fit differences are more readily detected in this application because an additional epoch adds an entirely new set of parameters. Incorporating more epochs further increased model fit (Table 3), albeit with diminishing returns, but it is clear that there are limits to the flexibility that can be incorporated in phylogeographic reconstructions, which represent inherently data-sparse inferences. In this respect, it is interesting to note that approaches are available to share information across epochs while still allowing for the detection of differences among them (Suchard et al. 2003). This can be achieved by specifying hierarchical priors, both on standard rate parameters (Edo-Matas et al. 2011) as well as, more recently, on the rate indicators in a BSSVS procedure (Cybis et al. 2013). Assuming that phylogeography represents a major application for the epoch model, further research is needed to explore these approaches as well as other sparse parameterizations of discrete dispersal processes.

The two data sets we examine represent examples with clear prior hypotheses that correspond to fixed transition times. It may also be of interest to apply the model when the transition times are unknown. Although it would be straightforward to estimate the time of a fixed number of epoch transition in our MCMC framework, estimating the number of epochs may be more challenging. However, our previous experience with change-point processes in phylogenetics (Suchard, Weiss, et al. 2003) suggests that it should be possible to introduce prior distributions over these quantities and jointly infer them when uncertainty remains in their specification. Instead of trying to identify the most appropriate discretization of time, one may potentially use epoch modeling to approximate more complex functions of time. Rodrigo et al. (2008) outline two approaches to allow rate matrices to vary as a function of time, one of which approximates that function by partitioning time into a fine grid of intervals. This is likely to be a computationally expensive avenue, but its performance will mostly depend on the availability of dense sampling throughout time (Rodrigo et al. 2008).

An interesting direction for further research using the epoch model is to couple epoch-specific parameters to external covariates. For the within-host HIV data sets we analyze here, it may be interesting to ask whether ω varies with changes in CD4 + counts or viral load. In our Bayesian framework, we can address this question through formulating a hierarchical phylogenetic model (Edo-Matas et al. 2011) over the epoch-specific parameters, considering the covariates as fixed-effects. We can further estimate the posterior probabilities of all possible linear models that may or may not include the covariates using Bayesian stochastic search variable selection (Kuo and Mallick 1998). In addition to external covariates, we can also consider connecting the evolutionary parameters in an epoch model to population size parameters in nonparametric models of population size change through time, such as the Bayesian skyline (Drummond et al. 2005), skyride (Minin et al. 2008), and skygrid models (Gill et al. 2013).

By adding an additional layer of complexity to our evolutionary models and inference framework, we further increase the computational demands in a field that is already computationally intensive. Our Bayesian approach integrates over all possible evolutionary scenarios, which is challenging for a large number of sequences even when specifying the simplest of evolutionary models. To mitigate the additional burden imposed by our epoch time-discretization and the operations involved, we have implemented our model in the high-performance BEAGLE library allowing us to perform the calculations on GPU architectures. Although this has proven extremely useful, in particular for large state-space models such as codon substitution models (Suchard and Rambaut 2009), more research is needed to further stretch the limits of practical computational restrictions.

In summary, our work has extended the phylodynamic framework with a model that is capable of quantifying and testing temporal heterogeneity in discrete state transition processes, which is proving useful to detect changing selective dynamics in rapidly evolving viral populations as well as fluctuations in historical circulation dynamics.

## Software Availability

BEAST source code is freely available at http://code.google.com/p/beast-mcmc/ (last accessed March 18, 2014) under the terms of GNU LGPL license. Compiled, ready-to-use binaries targeting major platforms can be obtained from http://beast.bio.ed.ac.uk (last accessed March 18, 2014). The BEAGLE library is free, open-source software licensed under the GNU LGPL. Both the source code and binary installers are available from www.code.google.com/p/beagle-lib/. SPREAD is licensed under the GNU Lesser GPL, and its source code is freely available at https://github.com/phylogeography/SPREAD (last accessed March 18, 2014). Compiled, runnable packages are hosted at http://rega.kuleuven.be/cev/ecv/software/spread (last accessed March 18, 2014).

## Supplementary material

Data available from the Dryad Digital Repository: http://datadryad.org/resource/doi:10.5061/dryad.qp747.

## Funding

The research leading to these results has received funding from the European Research Council under the European Community's Seventh Framework Programme (FP7/2007-2013) under Grant Agreement no. 278433-PREDEMICS and ERC Grant agreement no. 260864, the Wellcome Trust (grant no. 092807), the National Science Foundation (DMS 1264153), and the National Institutes of Health (R01 HG006139). The National Evolutionary Synthesis Center (NESCent) catalyzed this collaboration through a working group (NSF EF-0423641).
